# The Diagnostic Value of Lung Ultrasound in Bronchopulmonary Dysplasia Among Preterm Infants with Gestational Age ≤ 28 Weeks

**DOI:** 10.3390/diagnostics16152474

**Published:** 2026-08-05

**Authors:** Haifeng Zong, Bingchun Lin, Xueyu Chen, Yichu Huang, Jingyu Song, Hongyan Sun, Qingling Li, Sue Zhang, Chuanzhong Yang

**Affiliations:** 1 Neonatal Intensive Care Unit, Shenzhen Maternity and Child Healthcare Hospital, Women and Children’s Medical Center, Southern Medical University, Shenzhen 518028, China; zonghf123@smu.edu.cn (H.Z.); pureice1998268@126.com (B.L.); snowvsrain@smu.edu.cn (X.C.); huangyichu369@163.com (Y.H.); songjingyu1995103@163.com (J.S.); sunhy77@163.com (H.S.); qingling032@163.com (Q.L.); 2 Shenzhen Key Laboratory of Birth Defect Prevention and Control, Shenzhen 518028, China

**Keywords:** lung ultrasound, bronchopulmonary dysplasia, diagnosis, extremely premature infants, characteristic

## Abstract

**Objective**: The aim of this study was to explore the diagnostic value of lung ultrasound (LUS) for bronchopulmonary dysplasia (BPD) in extremely premature infants. **Methods**: This was a prospective observational cohort study in which infants with gestational age (GA) ≤ 28^+6^ weeks were included. LUS was performed at least once a week until 36 weeks of postmenstrual age. The LUS characteristics of infants with moderate–severe BPD were compared with those of infants with non–mild BPD. **Results**: A total of 114 infants were included, of which 69 (60.5%) had non–mild BPD, and 45 (39.5%) had moderate–severe BPD. The mean GA and birth weight of infants with non–mild BPD and moderate–severe BPD were 27.0 ± 1.4 and 26.3 ± 1.6 weeks and 969 ± 184 and 802 ± 228 g, respectively. The proportions of rough pleural lines, fused B-lines, patch-like anechoic appearance on the pleural surface, subpleural speckled hyperechoic appearance, lung consolidation (≥0.5 cm), fuzzy or invisible A-lines, and pleural insect erosion (PIE)-like changes in the moderate–severe BPD group were significantly greater than those in the non–mild BPD group (100% vs. 37.7%, 88.9% vs. 13.0%, 84.4% vs. 7.2%, 73.3% vs. 8.7%, 80.0% vs. 11.6%, 86.7% vs. 10.1%, and 75.6% vs. 5.8%, respectively; *p* < 0.001). In evaluating moderate–severe BPD, rough pleura had 100.0% (95% CI: 0.921–1.000) in sensitivity, 63.4% (95% CI: 0.518–0.736) in PPV, and 62.3% (95% CI: 0.505–0.728) in specificity. PIE-like changes had 75.6% (95% CI: 0.613–0.858) in sensitivity, 89.5% (95% CI: 0.759–0.958) in PPV, and 94.2% (95% CI: 0.860–0.977) in specificity. Pleura surface irregular patch-like anechoic had 84.4% (95% CI: 0.712–0.923) in sensitivity, 88.4% (95% CI: 0.755–0.949) in PPV, and 92.8% (95% CI: 0.841–0.969) in specificity. Subpleural speckled hyperechogenicity had 73.3% (95% CI: 0.592–0.840) in sensitivity, 84.6% (95% CI: 0.704–0.930) in PPV, and 91.3% (95% CI: 0.824–0.960) in specificity. **Conclusions**: LUS is a valuable tool for evaluating and diagnosing BPD.

## 1. Introduction

Bronchopulmonary dysplasia (BPD) is the most common morbidity in extremely preterm infants and is associated with long-term impairment of lung function [1]. The pathophysiology of BPD involves disrupted alveolarization, vascular remodeling, and airway alterations, leading to a spectrum of histopathological changes from alveolar simplification to interstitial fibrosis [2]. The diagnosis of BPD is based on the need for respiratory support at 36 weeks postmenstrual age (PMA), which does not reflect the underlying pathological changes. This diagnostic approach highlights the need for more objective tools to assess lung morphology and function in this vulnerable population [3].

Imaging plays a pivotal role in characterizing the structural abnormalities of BPD. Chest radiography, magnetic resonance imaging (MRI) and computed tomography (CT) can detect early ventilation defects, gas exchange abnormalities, airway alterations and impaired alveolar development [4,5]. Chest radiography has limited ability to characterize BPD. CT can provide details of the lungs for cysts, opacities and tracheobronchomalacia [6]. MRI plays a key role in providing CT-like imaging of the lungs, heart and vasculature without radiation [7]. Both CT and MRI require sedation or general anesthesia and cannot be performed at the bedside. Lung ultrasound (LUS) has emerged as a valuable point-of-care tool in neonatology and can guide surfactant therapy and predict outcomes [8]. LUS can be performed at the bedside without radiation and does not require sedation or anesthesia. In pediatric populations, LUS has demonstrated high diagnostic accuracy for various respiratory conditions. Recent studies have confirmed the usefulness of LUS in monitoring children with community-acquired pneumonia and in the early identification of those at risk of clinical deterioration. The abnormalities found on LUS can be quantified using scoring systems, and severity can be correlated with clinical outcomes [9,10].

Some studies have also described the imaging characteristics of LUS in BPD diagnosis [11,12,13,14]. However, the diagnostic criteria for BPD in those studies were on the basis of oxygen dependence 28 days after birth. In the current study, the GA of the infants was ≤28^+6^ weeks, and the diagnosis was based on the need for respiratory support at 36 weeks PMA. The aim was to outline the LUS features of non–mild and moderate–severe BPD.

## 2. Methods

### 2.1. Study Design

This prospective, single-center, observational cohort study was conducted at the neonatal intensive care unit (NICU) of Shenzhen Maternity and Child Healthcare Hospital, Women and Children’s Medical Center, Southern Medical University, from November 2023 to August 2024. This observational cohort study was approved by the Shenzhen Maternity and Child Healthcare Hospital Institutional Ethical Committee (No. SFYLS2023048), and informed consent was obtained from the parents.

### 2.2. Subjects

All infants with GA ≤ 28^+6^ weeks were included. The exclusion criteria included death before discharge, major congenital and chromosomal malformations, neuromuscular diseases and congenital malformation of the airway. BPD was evaluated by the definition of National Institute of Child Health and Human Development (NICHD) [3]. The definition of BPD depends on the supplemental oxygen and respiratory support at 36 weeks PMA.

### 2.3. Instrumental Examination

LUS was performed with a commercial transportable US device (M9, Mindray, Shenzhen, China) with a linear array probe (4–12 MHz) in B mode.

### 2.4. LUS Scanning

Each of the infants underwent LUS at least once a week (typically on days 7, 14, 21, 28 of life, and then weekly until 36 weeks PMA). For the statistical analysis in this study, we used the LUS findings at 36 weeks PMA to compare with the final BPD severity classification. LUS was scanned by a skilled operator who was blinded to the detailed information of the infants. All the images were copied for the assessment of BPD. Two evaluators blinded to the clinical information were responsible for assessing each image. Disagreements were resolved by consensus. The interobserver agreement was assessed using Cohen’s kappa coefficient.

The baby was in a prone position, lateral or supine. A linear array probe was used. Each lung field was divided into three areas (anterior, lateral and posterior areas) through the parasternal, anterior and posterior axillary lines. LUS scanning was performed in longitudinal and transverse orientations, and harmonics were not chosen. The probe was perpendicular to the chest wall.

### 2.5. LUS Characteristics of BPD

We summarized the LUS features of BPD with different severities and compared the specific signs of non–mild BPD with those of moderate–severe BPD.

(1) Pleural line abnormalities: The pleural line was assessed for thickness, roughness, and clarity. A pleural line was considered “obviously thick, rough, and fuzzy” when it appeared widened, irregular in contour, and poorly defined compared with the normal thin, smooth, and echogenic pleural line. Pleural insect erosion (PIE)-like changes were defined as discontinuous and intermittent disruption of the pleural line, resembling a moth-eaten appearance.
(2) lines: Horizontal echogenic lines parallel to the pleural line, representing reverberation artifacts from the pleural surface. A-lines were classified as “fuzzy, not clear, or invisible” when they were partially or completely obliterated by B-lines or other artifacts.
(3) B-lines: Vertical hyperechoic artifacts arising from the pleural line, extending to the bottom of the screen without fading. B-lines were classified as “multiple, partial or completely fused” when they were too numerous to count and coalesced into confluent bands, indicating significant interstitial or alveolar fluid accumulation.
(4) Lung consolidation: Defined as a tissue-like echogenicity region with air bronchograms (hyperechoic punctate or linear artifacts within the consolidation). Regarding the differentiation between atelectasis and pneumonia on LUS, atelectasis was differentiated from pneumonia/consolidation based on the following criteria: (1) the presence of dynamic air bronchograms (moving with respiration) was more suggestive of pneumonia, whereas static air bronchograms were more consistent with atelectasis; (2) the presence of associated pleural line abnormalities or B-lines in surrounding lung zones favored pneumonia; and (3) atelectasis typically resolved or improved with positive pressure ventilation or position changes, whereas pneumonic consolidation was more persistent.
(5) Pleural effusion: Defined as an anechoic space between the parietal and visceral pleura.
(6) Irregular patch-like anechoic or weakly echoic appearances on the pleural surface: Defined as irregular, well-demarcated areas of absent or reduced echogenicity on the pleural surface, with no detectable blood flow on color Doppler.
(7) Subpleural speckled or patch-like hyperechogenicity: Defined as scattered, punctate or patchy bright echoes located immediately below the pleural line, which may correspond to microcysts, fibrosis, or inflammatory changes in the subpleural region.

All findings were assessed in each of the six lung zones. The presence of each finding was recorded as a binary variable (present/absent) for each infant. The severity of LUS findings was not scored quantitatively in this study; rather, we compared the prevalence of each finding between the non–mild BPD and moderate–severe BPD groups.

### 2.6. Statistical Analysis

Statistical analysis was performed with SPSS Version 23 software (IBM Corporation, Armonk, NY, USA). Continuous variables conforming to normal distribution were expressed as mean ± standard deviation, while those with non-normal distribution were presented as median (interquartile range, IQR). Categorical variables were described as counts (percentages). Between the non-to-mild BPD group and the moderate-to-severe BPD group, chi-square tests were used to compare differences in categorical variables; Fisher’s exact test was employed when the expected frequency was <5 in more than 20% of cells. For continuous variables with normal distribution and homogeneous variance, an independent samples *t*-test was used for intergroup comparison. Non-normally distributed continuous variables were compared using the Mann–Whitney U test. Cohen’s k coefficient was also used to assess the interobserver variability. The sample size was estimated as follows. In the year prior to this research, the incidence of BPD was approximately 40% in infants ≤28^+6^ weeks’ gestation in the NICU. A random sampling formula was used for sample size estimation, with α = 0.05 and δ = 0.1. A design effect of 1.1 yielded an effective sample size of 102. Considering a 10% dropout rate, the final enrolled sample size was 113 neonates. All statistical tests were two-sided, and a *p*-value < 0.05 was considered statistically significant.

## 3. Results

A total of 129 eligible infants with GA ≤ 28^+6^ weeks were admitted to our NICU during this period. Fifteen (11.6%) infants were excluded from the study because of death before 36 weeks PMA. Finally, 114 infants were included: 69 (60.5%) with non–mild BPD and 45 (39.5%) with moderate–severe BPD. The basic characteristics of the infants are described in Table 1. Those who developed moderate–severe BPD had lower birth weight, lower rate of cesarean section, lower rate of full course of antenatal steroids, lower Apgar score, and higher rate of hemodynamically significant patent ductus arteriosus (hsPDA) and pulmonary surfactant therapy than those who did not develop moderate–severe BPD.

**Figure 1:**
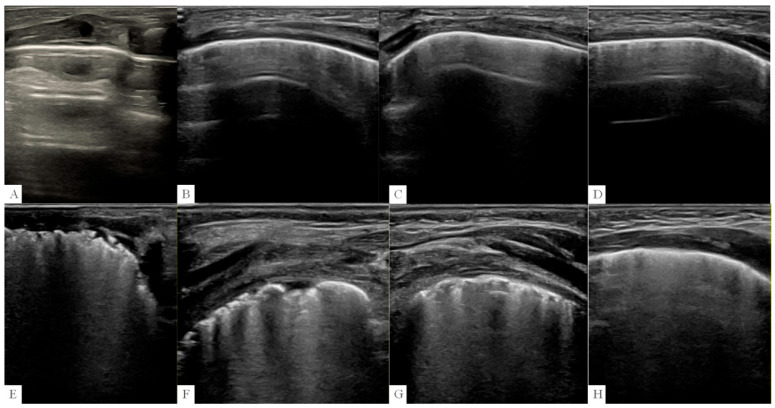
The pleural line of non–mild and moderate–severe BPD infants. (**A**–**D**). In infants with non–mild BPD, the pleural line is slightly thick, rough, and fuzzy. (**E**–**H**). In infants with moderate–severe BPD, the pleural line is obviously thick, rough, and fuzzy. Notably, the pleural line is discontinuous and intermittent, similar to pleural insect erosion (PIE)-like changes.

LUS imaging results of neonates with moderate–severe BPD are characteristic. Table 2 shows the results of the LUS findings in the non–mild BPD and moderate–severe BPD groups. In the non–mild BPD group, the pleural line is slightly thick, rough, and fuzzy (Figure 1A–D). Compared with that in the non–mild BPD group, the pleural line in the moderate–severe BPD group was obviously thick, rough, and fuzzy (37.7% vs. 100%, *p* < 0.001) (Figure 1E–H). Notably, the pleural line is discontinuous and intermittent, similar to pleural insect erosion (PIE)-like changes (5.8% vs. 75.6%, *p* < 0.001). Table 3 shows the reliability of each of the LUS features in evaluating moderate-to-severe BPD.

**Figure 2:**
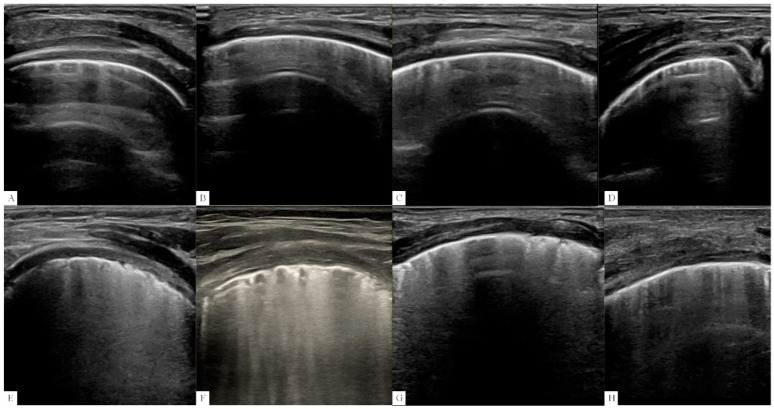
The B-lines and A-lines of non–mild and moderate–severe infants. (**A**–**D**). In infants with non–mild BPD, the B-lines are tiny and sparse and lack obvious fusion, and the A-lines are almost clearly visible. (**E**–**H**). In infants with moderate–severe BPD, the B-lines are multiple and difficult to count and are partial or even completely fused, and the A-line is fuzzy, unclear, and even invisible.

**Figure 3:**
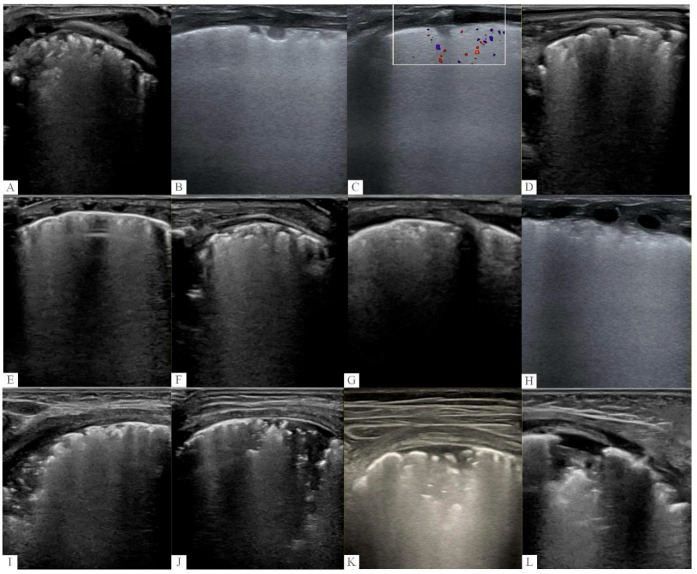
Other LUS appearances of infants with moderate–severe BPD. (**A**–**D**). In infants with moderate–severe BPD, irregular patch-like anechoic or weakly echoic appearances on the pleura surface were observed, and no blood flow was detected in the anechoic areas on color Doppler. (**E**–**H**). Scattered speckled or patch-like hyperechogenicity below the pleura was observed. (**I**,**J**). Lung consolidation accompanied by air bronchograms (>0.5 cm) was observed. (**K**,**L**). Consolidation accompanied by pleural effusion was also found in a few patients, although the difference was not statistically significant.

In the non–mild BPD group, the B-lines were tiny, sparse and without obvious fusion and had little effect on the A-lines, and the A-lines were almost clearly visible (Figure 2A–D). In the moderate–severe BPD group, multiple B-lines were difficult to count and were partial or even completely fused (88.9% vs. 13.0%, *p* < 0.001), significantly affecting the A-line, and the A-line was fuzzy, unclear, and even invisible (Figure 2E–H) (86.7% vs. 10.1%, *p* < 0.001). Furthermore, in the moderate–severe BPD group, irregular patch-like anechoic or weakly echoic appearances on the pleura surface were observed, and no blood flow was detected in the anechoic areas on color Doppler (Figure 3A–D) (84.4% vs. 7.2%, *p* < 0.001). Scattered, speckled or patchy hyperechogenic appearance below the pleura was observed (Figure 3E–H) (73.3% vs. 8.7%, *p* < 0.001). Lung consolidation accompanied by air bronchograms (>0.5 cm) was also observed (Figure 3I,J) (80.0% vs. 11.6%, *p* < 0.001). Interestingly, consolidation accompanied by pleural effusion was also found in a few patients, although the difference was not statistically significant. (Figure 3K,L) (6.7% vs. 0, *p* = 0.059). The interobserver agreement of two evaluators for the LUS images was high (K = 0.90, 95% CI: 0.86–0.95). Table 2 shows the difference in LUS findings between the non–mild BPD group and the moderate–severe BPD group.

In infants who underwent serial LUS examinations, the characteristic findings of early BPD (before 3–4 weeks)—including thickened and rough pleural lines, fused B-lines, fuzzy/invisible A-lines and lung consolidation changes—became progressively more apparent over the course of the hospitalization. About 3–4 weeks later, irregular patch-like anechoic or weakly echoic appearances on the pleura surface, scattered patch-like hyperechogenicity below the pleura and the pleural line PIE-like changes gradually occur, although their extent varied among individuals.

## 4. Discussion

Our results demonstrate that compared with infants with non–mild BPD, infants with moderate–severe BPD have unique LUS features, including thick, rough, fuzzy and discontinuous pleural lines; similar PIE-like changes; multiple, partial or even completely fused B-lines, irregular patch-like anechoic or weakly echoic appearances on the pleura surface; speckled or patchy hyperechogenic appearance below the pleura; and lung consolidation accompanied by air bronchograms. This appearance underscores the potential of LUS as a tool for risk stratification.

In the NICU, bedside chest radiography is a common imaging method. However, its value in BPD is limited. Lung CT and MRI have good diagnostic and predictive values [15]. Nevertheless, CT scans revealed radiation. Both CT and MRI require transportation, which is risky for patients with severe BPD. LUS is a modality that has gained increasing popularity for the study of neonatal diseases [8]. LUS is portable and does not involve radiation and can dynamically monitor patients at the bedside.

The pathological features of BPD are airway damage, chronic inflammation of the lung, fibrosis and localized emphysema [16]. Therefore, exploring the diagnosis and assessment of BPD is particularly important. Before the development of LUS, some studies focused on the association between BPD development and the specific findings of ultrasonography. Avni et al. described the persistence of retro-diaphragmatic hyperechogenicity at 28 days of life in all infants who later developed BPD, whereas this finding was absent in 95% of neonates who did not progress to BPD [17]. Similar results were observed by Pieper et al., who also identified day 9 as the earliest day of assessment with the highest predictor values for BPD development [18].

In the current study, although B-lines were present in both the non–mild BPD group and the moderate–severe BPD group, the B-line distributions were more diffuse or fused, significantly affecting the A-line, and the A-line was not clear and was even invisible in the moderate–severe BPD group. B-lines are often observed in the lungs, such as in pneumonia, pulmonary edema, and pulmonary fibrosis [19]. The causes of B-lines in infants with BPD are rather complex. BPD is caused by an abnormal reparative response to antenatal and postnatal damage to preterm lungs [16]. MRI has shown that infants with BPD have increased lung water and are susceptible to alveolar flooding and gravity-induced collapse in the dependent areas of the lung [20]. Infants with moderate–severe BPD had increased lung water in dependent areas. The heavy lung water burden led to high hydrostatic pressure, alveolar collapse or the induction of flooding of the alveolar compartment. This may be one of the reasons for the increase in B-lines, although this remains speculative and requires confirmation in future studies. In addition, infants with BPD are prone to pneumonia and pulmonary infection. Fibrous exudation and neutrophils and macrophages in the alveoli can also cause diffuse B-lines. In an animal model, the alveolar wall and septum were unevenly thickened, and inflammatory cell infiltration was characterized by significantly increased pulmonary macrophages, collagen and fibrin deposition in the alveolar septa [21]. Similarly, Liu et al. reported that the most common LUS manifestations of BPD are scattered or fused B-lines [12].

The specific ultrasound findings revealed irregular patch-like anechoic or weakly echoic appearances on the pleural surface, and no blood flow was detected in the anechoic areas on color Doppler. These findings are different from those observed in lung consolidation. The same LUS performance was also observed in animal models [21]. Previous research also revealed small cysts above the diaphragm in moderate–severe BPD, which appeared anechoic with surrounding hyperechogenicity without air bronchogram and blood flow and were located above the diaphragmatic surface [14]. Another study used “debris” signs to diagnose BPD: subpleural weak echo areas with fragment-like strong echoes [11]. Based on pathological findings from previous animal and human studies, we hypothesize that these ultrasound findings may correspond to pulmonary fibrosis and thickening of the alveolar wall and septum, although direct histopathological correlation in our patient population was not performed [21].

Scattered speckled or patch-like hyperechogenicity below the pleura was observed in the current study. Liu et al. also described dot-like hyperechoic patterns situated in the fields of alveolar-interstitial syndrome, which were a specific sign of BPD on LUS and were seen in 53.1% of infants with BPD. They reported that specific hyperechoic patterns on LUS may correspond to pulmonary vesicles or pulmonary cysts in infants with BPD [12]. The same LUS performance was also observed in animal models [21]. Another specific ultrasound finding revealed PIE-like changes, characterized by a discontinuous and intermittent pleural line, with a moth-eaten appearance. Liu et al. reported PIE-like changes in 62.5% of infants with late-stage BPD [12]. We hypothesize that these pathological changes may be related to fibrosis in the alveolar septa and inflammatory cell infiltration affecting the pleura. This hypothesis warrants further investigation in future studies with histopathological correlation.

In recent years, semiquantitative LUS scoring systems have been developed to standardize the assessment of pulmonary abnormalities and to predict clinical outcomes. Several studies have demonstrated that LUS scores correlate with BPD severity and can predict the development of moderate–severe BPD [13,22,23]. Han et al. reported that the diagnostic accuracy of LUS scoring for BPD improved progressively from the first through the third weeks of life, with an AUROC of 0.92 by day 21 [24]. These findings support the integration of serial LUS assessments into neonatal care protocols for early, non-invasive BPD diagnosis and risk stratification. While our study did not employ a formal scoring system, the specific qualitative findings we identified may serve as the foundation for developing a BPD-specific LUS scoring system in future studies.

According to the results of this study, lung consolidation accompanied by air bronchograms is the common LUS manifestation of moderate–severe BPD. The extent of its lesion often exceeds 0.5 cm. In the non–mild BPD group, lung consolidation exceeding 0.5 cm was rare. Interestingly, in some consolidated areas of moderate–severe BPD, local pleural effusion may occur concurrently, although the incidence is very low. Similarly, more consolidations and pleural line abnormalities have been observed by other researchers in infants with BPD [11].

While we did not perform formal statistical correlation analyses between LUS findings and other diagnostic tests, we observed that infants with more severe LUS abnormalities consistently required higher levels of respiratory support. These clinical observations support the potential utility of LUS in monitoring disease progression, although prospective studies with systematic correlation are needed to validate these findings.

We acknowledge several research limitations in our study. First, the number of neonates included in this study was relatively small. Second, we did not perform correlation analyses between LUS findings and laboratory parameters (such as inflammatory markers or blood gas analysis) or oxygen requirements, which would have provided additional insight into the clinical significance of the LUS findings. Future studies incorporating such correlations are warranted. Third, the pathogenesis of BPD is multifactorial and includes pulmonary vascular, atelectasis, cystic lesions and consolidation. Therefore, the assessment of moderate–severe BPD involves not only the lungs but also circulation. This is one of the disadvantages of ultrasound. However, LUS is portable and does not involve radiation and can be used to dynamically monitor patients with BPD. In addition, the images behind the diaphragm were not scanned in this study. More multicenter studies are needed in the future.

## 5. Conclusions

In conclusion, LUS could provide characteristic ultrasound imaging in patients with moderate–severe BPD. This finding may aid clinicians in the diagnosis of BPD.

## Figures and Tables

**Table 1:** Baseline characteristics of the infants.

	Non–Mild BPD (*n* = 69)	Moderate–Severe BPD (*n* = 45)	*p*-Value
GA (weeks)	27.0 ± 1.4	26.3 ± 1.6	0.135
Male (%)	47 (68.1%)	31 (68.9%)	0.931
Cesarean section (%)	47 (68.1%)	21 (46.7%)	0.023
Birth weight (g)	969 ± 184	802 ± 228	<0.001
PROM > 18 h (%)	58 (84.1%)	32 (71.1%)	0.097
Antenatal steroids, full course (%)	60 (87.0%)	30 (66.7%)	0.009
Apgar score 1 min	8 (7, 9)	5 (5, 8)	<0.001
Apgar score 5 min	9 (9, 10)	9 (7, 9)	<0.001
hsPDA	13 (18.8%)	17 (37.8%)	0.025
IVH grade III–IV (%)	2 (2.9%)	4 (8.9%)	0.211
NEC or SIP	3 (4.3%)	3 (6.7%)	0.679
pulmonary surfactant therapy (%)	43 (62.3%)	37 (82.2%)	0.023

Values are expressed as mean ± standard deviation, median (25th; 75th centile), or number (%). BPD, bronchopulmonary dysplasia; GA, gestational age; hsPDA, hemodynamically significant patent ductus arteriosus; IVH, intraventricular hemorrhage; NEC, necrotizing enterocolitis; PROM, premature rupture of membrane; SIP, spontaneous intestinal perforation.

**Table 2:** Lung ultrasound findings in the non–mild BPD and the moderate–severe BPD.

Lung Ultrasound Findings	Non–Mild BPD (*n* = 69)	Moderate–Severe BPD (*n* = 45)	*p*-Value
Pleural line obviously thick, rough, fuzzy (%)	26 (37.7%)	45 (100%)	<0.001
Pleural insect erosion (PIE)-like changes (%)	4 (5.8%)	34 (75.6%)	<0.001
Irregular patch-like anechoic or weakly echoic on the pleura surface (%)	5 (7.2%)	38 (84.4%)	<0.001
Subpleural speckled or patch-like hyperechogenicity (%)	6 (8.7%)	33 (73.3%)	<0.001
A-line fuzzy, not clear, even invisible (%)	7 (10.1%)	39 (86.7%)	<0.001
Multiple, partial or complete fused B-lines (%)	9 (13.0%)	40 (88.9%)	<0.001
Lung consolidation (≥0.5 cm) (%)	8 (11.6%)	36 (80.0%)	<0.001
Pleural effusion (%)	0 (0%)	3 (6.7%)	0.059

BPD, bronchopulmonary dysplasia.

**Table 3:** The reliability of the lung ultrasound findings in evaluating moderate-to-severe BPD.

LUS Features	Sensitivity (95%CI)	Specificity (95%CI)	PPV (95%CI)	NPV (95%CI)
Pleural line obviously thick, rough, fuzzy	100.0 (92.1~100.0)	62.3 (50.5~72.8)	63.4 (51.8~73.6)	100.0 (91.8~100.0)
Pleural insect erosion (PIE)-like changes	75.6 (61.3~85.8)	94.2 (86.0~97.7)	89.5 (75.9~95.8)	85.5 (75.9~91.7)
Irregular patch-like anechoic or weakly echoic on the pleura surface	84.4 (71.2~92.3)	92.8 (84.1~96.9)	88.4 (75.5~94.9)	90.1 (81.0~95.1)
Multiple, partial or complete fused B-lines	88.9 (76.5~95.2)	87.0 (77.0~93.0)	81.6 (68.7~90.0)	92.3 (83.2~96.7)
Lung consolidation (≥0.5 cm)	80.0 (66.2~89.1)	88.4 (78.8~94.0)	81.8 (68.0~90.5)	87.1 (77.3~93.1)
Subpleural speckled or patch-like hyperechogenicity	73.3 (59.2~84.0)	91.3 (82.4~96.0)	84.6 (70.4~93.0)	84.0 (74.2~90.6)

BPD, bronchopulmonary dysplasia; CI, confidence interval; LUS, lung ultrasound; NPV, negative predictive value; PPV, positive predictive value. Note: Reliability data are reported with 95% confidence intervals.

## Data Availability

The data presented in this study are available on request from the corresponding author due to ethical restriction.
